# ‘Striving to achieve control’. Registered nurses’ experiences of palliative care quality during the COVID-19 pandemic – a qualitative study

**DOI:** 10.1186/s12904-024-01644-8

**Published:** 2025-01-23

**Authors:** Tuva Sandsdalen, Ann Karin Helgesen, Vigdis Abrahamsen Grøndahl, Carina Bååth, Maria Larsson, Christina Melin Johansson, Cecilia Olsson, Maria Tillfors, Jane Österlind, Reidun Hov, Marie Dahlen Granrud

**Affiliations:** 1 Department of Health and Nursing Sciences, Faculty of Health and Social Sciences, University of Inland Norway, Elverum, Norway; 2 Faculty of Health, Welfare and Organization, Østfold University College , Halden, Norway; 3 Department of Health Sciences, Faculty of Health Science and Technology, Karlstad University, Karlstad, Sweden; 4 Department of Bachelor’s in Nursing, Lovisenberg Diaconal University College, Oslo, Norway; 5 Department of Social and Psychological Sciences, Faculty of Arts and Social Sciences, Karlstad University, Karlstad, Sweden; 6 Department of Healthcare Sciences/Nursing, Mid Sweden University, Östersund, Sweden; 7 Department of Health Care Sciences, Marie Cederschiöld University, Stockholm, Sweden; 8 Centre for Development of Institutional and Home Care Services, Inland (Hedmark), Hamar Municipality, Norway; 9 Department of Social Sciences and Guidance, Faculty of Health and Social Sciences, University of Inland Norway, Elverum, Norway

**Keywords:** COVID-19, Hospice and palliative care nursing, Nursing care, Palliative care, Quality of health care, Registered nurses’ experience

## Abstract

**Background:**

Providing quality palliative care during a pandemic was challenging. Both specialist and community healthcare services cared for patients that faced life-threatening illness and who were influenced by the restrictions of the COVID-19 pandemic. Little knowledge has yet been provided on how registered nurses (RNs) experienced the palliative care quality during the COVID-19 pandemic. The aim of this study was to explore RN’s experiences of providing palliative care quality during the COVID-19 pandemic.

**Methods:**

This qualitative study had a descriptive design. Semi-structured individual interviews were conducted between November 2021 and January 2022 with 18 RNs who worked in intensive care units in hospitals, dementia care or palliative care units in nursing homes in Norway during the pandemic. Data were analysed by using qualitative content analysis. The study was conducted and reported according to the COREQ’s checklist.

**Results:**

Analysis of the data resulted in an overall theme: ‘Striving to achieve control’. This theme comprised six categories: (1) when the toolbox does not fit; (2) protective equipment—social distance and opportunities for closeness; (3) unpredictable workday; (4) the right person to the right assignment at the right time; (5) presence and absence of relatives and friends; and (6) situations that required creativity. RNs had various experiences regarding how the quality of care was perceived; being worse, preserved, or in some cases even better than before the pandemic.

**Conclusions:**

The provision of quality palliative care was experienced by RNs as challenging during the pandemic. The pandemic forced them to be creative and to strive for control to provide the best palliative care possible given the situation. The results of this study may contribute to important knowledge for leaders, policy makers and RNs to learn from the COVID-19 pandemic and planning for future pandemics or crises. Especially to optimise factors perceived by RNs to be important for the palliative care quality, related to the specific situation and care context, to include the perspectives of those involved and take into consideration the time perspective of the pandemic.

**Supplementary Information:**

The online version contains supplementary material available at 10.1186/s12904-024-01644-8.

## Background

Providing palliative care is challenging when facing a pandemic, with many people in need of care from an overloaded healthcare system. However, failing to do so could have serious consequences such as extended human suffering, mistrust of healthcare personnel among patients and their relatives, burn out and post-traumatic stress disorder among health care personnel [1].

Palliative care should be offered to all patients in need, from the time of diagnosis with life-threatening illness until death, including support to relatives and bereavement care [2–4]. Palliative care involves the prevention and alleviation of physical, psychosocial and spiritual suffering with the aim of achieving the best possible quality of life [3]. The concept of palliative care also includes end - of - life care, that more specifically involves comprehensive care for dying patients in the last hours, days or weeks of life [4] and that the main goal of the care changes from being life-prolonging to be relieving. COVID-19 infections led to life-threatening illness in some patients, especially older persons with pre-existing chronic illnesses. Many elderly persons at risk are those living in nursing homes, being frail and depended upon care, who might be exposed to COVID-19 from staff, visitors or other residents, and unable to isolate themselves [5]. Challenges in caring for these patients have been seen internationally and in Norway related to a lack of equipment for infection control, high workload for the staff, fear of be infected and spreading the virus, and emotional burden for the staff [6, 7]. In addition, challenges were related to comprehensive restrictions in all healthcare services, both in specialist healthcare and in the community-based services including restrictions regarding visits from relatives and friends and the use of personal protective equipment (PPE) [5, 6, 8].

Both specialist and community healthcare services have been involved in caring for patients with life threatening illness and were influenced by the restrictions of the pandemic. These healthcare services aim to provide quality care to all patients including those in need of palliative care [9, 10]. Quality care means that health services should be effective, safe, person-centred, timely, equitable, integrated and efficient [9, 10]. To provide quality palliative care, it is essential to implement the principles of palliative care [2, 3]. Palliative care should also be provided according to principles of person-centred care that involve providing care that responds to individual preferences, needs and values [10].

Regardless of the pandemic, major challenges have been described in palliative care both internationally and in Norway. Important challenges for quality of care and, especially, palliative care have been related to information, symptom control, continuity and cooperation between professions and levels of healthcare services [11, 12]. The importance of enhanced palliative care in community healthcare has been one of the most highlighted areas for improvement in several central government documents and reports evaluating palliative care [13–15] and has been further actualised during the pandemic.

Little knowledge has yet been provided of the quality of palliative care during the pandemic, more specifically how the registered nurses (RNs) experience the care for life-threateningly ill, patients. Such knowledge is important for learning from the COVID-19 pandemic, for handling future pandemics and giving quality palliative care for all who are in need.

Previous research has commonly focused on frontline nurses’ (nurses working in hospitals, e.g., emergency departments and intensive care units, and community services, e.g., quarantine facilities and medical centres) experiences during the pandemic and how these experiences affected them psycho-emotionally, physically, and ethically. A previous review about nurses in frontline experiences during the first six months of the COVID-19 pandemic showed that nurses experienced emotional and moral challenges related to fear and stress of being infected, for example, which had an impact on their ability to meet patients’ psychological and emotional needs, a reduced frequency of nursing tasks and concerns about the lack of procedures to guide their care [7]. Two national Norwegian reports have described nurses’ experiences of the first phase of the pandemic [6] along with case studies of experiences with the COVID-19 pandemic from nursing homes [8]. The results underlined a need for transferring responsibilities and work tasks from RNs to assistant nurses, an increased sense of togetherness [8], a lack of PPE in the beginning of the pandemic and challenges related to caring for patients’ and their relatives’ psychosocial needs [6, 8]. However, the research seems to be scarce regarding how RNs’ experience the influence of the pandemic on the quality of palliative care, and how their experiences changed during the pandemic, if at all. One study from intensive care units, medical care units and nursing homes found that healthcare personnel perceived that quality of palliative care provided was not in line with what they perceived to be important for the patients [16]. Two studies conducted in palliative care units, however, found that healthcare personnel struggled to provide quality palliative care during the pandemic [17, 18]. Because of the restrictions, infection control routines and the lack of PPE, the healthcare personnel experienced ethical dilemmas in their ability to fulfil the patients’ preferences and last wishes [18]. More specifically, healthcare personnel experienced an altered relationship with patients and families, citing reduced communication and distance due to their use of protective equipment. They also experienced altered working dynamics due to relocations and a decreased use of healthcare resources that led to late referral to palliative care, late diagnoses and negative effects on symptom control. Lastly, healthcare personnel reported using strategies to improve the palliative care quality with digital communication and the development of protocols and procedures to the pandemic [17].

In sum, the research on RNs’ experiences of palliative care during the COVID-19 pandemic and their perspectives on what inhibits or promotes palliative care quality is sparse nationally and internationally. The aim of this study was to explore RNs’ experiences of palliative care quality during the COVID-19 pandemic, to fill this gap.

Research questions:

1. How did RNs experience providing palliative care during the pandemic?
2. Which factors promoted and inhibited the possibility to provide quality palliative care?

## Methods

### Design

A qualitative descriptive design was used to gain a deeper understanding of RNs’ experiences of providing palliative care during the pandemic [19]. The study followed the COREQ checklist for qualitative studies [20]. This study is a part of a larger multi-centre study, Palliative Quality Care COVID-19 (PaQC-C19), conducted in Sweden and Norway.

### Study setting and procedures

Palliative care is provided by various type of services, in specialist health services and in community care. Participants were included from both care contexts; two intensive care units in hospitals (ICU), one dementia care unit and one palliative care unit in two nursing homes in Norway that all provided palliative care during the COVID-19 pandemic. In Norway, RNs working in all healthcare services are required to hold basic palliative care competence acquired from their basic professional education [2]. However, RNs working in specialised palliative care services that mainly care for patients in palliative phases should have further education in palliative care [4].

Participants were included by the following criteria: RNs who have experienced caring for life-threatening ill patients during the COVID-19 pandemic in Norway. The leaders of the services were asked to recruit eligible participants, provide written information about the study, and schedule the time and place for the interviews.

### Participants

In total, 18 RNs with a variation in age and work experience were included in this study. Fifteen participants had expanded their position beyond their ordinary work positions and three had been relocated during the pandemic. Of these, relocations were both imposed and voluntary. Table 1 shows the participants’ characteristics.

**Table 1:** Background characteristic of the participants

*N* = 18	
	*n*
**Gender**	
Male	1
Female	17
**Age (years)**	
30–39	3
40–49	7
50–59	6
60–65	2
**Further education**	
Cancer nursing	5
Intensive care nursing	10
Dementia care nursing	1
None	2
**Workplace**	
Intensive care unit	10
Palliative care unit in a nursing home	5
Dementia care in a nursing home	3
**Work experiences in present unit (years)**	
5–10	13
11–20	5

### Data collection

A semi-structured interview guide was developed and discussed with all authors and two RNs with experience in palliative care nursing. The guide was used to ensure that the aim of the study was covered, and that each participant was asked to elaborate on the same topics [19]. The interview guide was pilot tested by conducting the interview within the research team and by interviewing two RNs. The pilot test did not lead to any amendments of the guide. Therefore, the two pilot interviews were included in the study.

The interview guide included questions related to demography and questions about providing palliative care quality during the pandemic. This study reports from the following main questions in the interview guide developed for this study (Supplementary file 1): What characterises (good) person-centred palliative care for you? What is your experience in providing palliative care during the COVID-19 pandemic? Follow-up questions were asked to obtain more detailed information of the RN’s experiences; for example, ‘Can you please tell me about situations where care was good/not so good’ and ´Which factors promoted and inhibited the possibility to provide good person-centred palliative care during the pandemic´ and ‘Can you please explain more about that…?’

Data were collected between November 2021 and January 2022. All interviews were individual and three of the authors (TS, MDG, AKH) collected 13 interviews. In addition, two master’s degree students collected five interviews, supervised by AKH. All the interviews took place at the workplace of the participants, according to their preferences. The interviews lasted from 20 to 58 min and were digitally recorded and transcribed verbatim.

### Data analysis

The interviews were analysed using qualitative content analysis as described by Graneheim and Lundman [21, 22]. The interviews were read several times by the first author to ensure that they were transcribed accurately and correctly. Then four of the authors (TS, AKH, VAG, MDG) read the interviews to familiarise themselves with the content and to acquire an overall understanding. Five authors (TS, AKH, VAG, RH, MDG) were assigned to carefully read 2–5 interview transcripts each and suggest meaning units which correspond with the aim of the study. The meaning units were identified, condensed, abstracted and labelled with a code. The codes were compared based on similarities and differences and sorted into five categories of manifested content and one overarching theme [21]. During the analysis, the six categories and the theme were discussed by the same five authors until a consensus was reached. The categories and theme were then discussed within the research group.

## Results

Analysis of the data resulted in an overall theme ‘striving to achieve control’. This theme is interpreted as the latent content of the manifested six categories that described the RNs’ experiences of providing palliative care during the pandemic and what factors the RNs’ experienced that promoted and inhibited the possibility to provide quality palliative care. The theme and categories are presented in Table 2.

**Table 2:** Overview of the overarching theme and the categories

Striving to achieve control					
When the toolbox does not fit	Protective equipment, social distance and opportunities for closeness	Unpredictable workday	The right person to the right assignment at the right time	Presence and absence of relatives and friends	Situations that required creativity

### Striving to achieve control

The RNs strived to achieve control to maintain the quality of palliative care despite the unpredictable work situation during the pandemic. The strive for control was expressed in the six categories described in Table 2.

Variations emerged with regards to how RNs experienced the impact of the COVID-19 pandemic on the quality of palliative care. Overall, RNs experienced that whether the quality of palliative care deteriorated, improved, or remained unchanged depended on the situation (what), the perspective (who) and the care context (where). The quality of care also depended on the time perspective (when), in that RNs’ experiences varied over time during the pandemic period. Some RNs experienced that it was difficult to provide the same quality palliative care that they were used to and that was in line with principles of person-centred palliative care. This was mainly because of the restrictions that followed the pandemic, which led them to strive for control to ensure palliative care quality. On the other hand, some RNs experienced that the quality of care was unchanged in that central elements of palliative care were maintained. Lastly, some RNs even experienced that the quality of care improved during the pandemic, since less time was spent on relatives and friends visiting the patients which led to more time spent with the patients. Additionally, in the ICU, RNs experienced that many of the patients with COVID-19 stayed longer than patients in the past, which led to a more predictable workday and the possibility of knowing the patients better.

### When the toolbox does not fit

The ‘toolbox’ experienced by RNs is comprised of internal factors like experience and knowledge and external factors such as guidelines and routines. The pandemic led to changes in both the patient group and the palliative care required by the patients. For example, patients with COVID-19 had a more rapid deterioration, unpredictable progression and a palliative care course with more violent symptoms and deaths; patients needed higher doses of medications, such as morphine, than some RNs were familiar with. These patients were also difficult for RNs to judge whether they were dying or would recover. Therefore, the usual routines and palliative care guidelines—the Norwegian revised version of the Liverpool Care Pathway (The last days of life)—could not automatically be applied to these patients. One RN from the nursing home stated:

‘…*the medication that we are used to give in the palliative phase was not enough…those who died…with COVID…it happened very quickly and…it was dramatic in some cases*’ (Interview 16).

Another RN from the ICU stated that this was especially related to the beginning of the pandemic:

‘*In the beginning*,* it was a lot of uncertainty. We didn’t know… how a COVID patient would react on a ventilator… now we started with placing the patients in prone position*,* it’s something new for me… they have to be turned over in the night*,* they must change positions every four hours… so it is… in a way*,* a new patient group*,* which has come up with new things*’ (Interview 10).

Also, some RNs experienced lack of competence when providing palliative care. Participants were concerned that when the usual toolbox/guidelines did not apply or were difficult to use, the patients did not receive quality palliative care. However, some RNs perceived this differently in that they were familiar with this pace, had the competence needed to handle similar patients, and were now transferring these competencies to the new group of patients with COVID. This was especially found in RNs who worked the ICU and with more years of work experience.

A lack of competence and experience with infection control and the use of protective equipment was experienced in the beginning of the pandemic. However, as the pandemic lasted, RNs experienced an increased competence related to patients with COVID, including infection control and handling the restrictions within the units, which led to feelings of security and confidence in handling potential pandemics in the future. However, the competence development and training in other areas beyond infection control remained on hold.

### Protective equipment: social distance and opportunities for closeness

RNs reported using various PPE depending on the patients’ situation, e.g., if patients were infected by COVID or were in quarantine and depending on the national or regional restrictions and the amount of infected persons in the country. Using PPE was perceived by RNs to influence the quality of care in different ways. RNs from both nursing homes and the ICU experienced that using PPE led to social distance between themselves and the patients. Using face masks, eye protection, full cover suit and cap reduced their ability to communicate with patients using their body language as facial expressions were not visible and verbal communication was reduced due to difficulties hearing and speaking through facial masks. As one RN in ICU expressed:

‘*The protective equipment provides a greater distance…I think so. It is more difficult to see the eyes through glasses and visors and… You are completely wrapped in. It is not easy to see the person inside. It gives a great distance’* (Interview 13).

When using PPE it was also perceived by RNs to be difficult for patients to know which nurse cared for them since they could not see their faces. RNs experienced that those patients reacted negatively when using the equipment and even became nervous, more agitated or delirious and sometimes worsened the patients’ condition. RNs experienced that this inhibited communication and talks, which they considered an important component of quality of palliative care. This was described by a RN in the nursing home:

‘*There was a lot of grief*,* both from the patient and me. I was supposed to comfort*,* but it was difficult because I was fully dressed in protective equipment. In other words*,* not being able to touch the patient—wearing gloves and mask and*,* you know—you lose a part of the look and facial expression. So*,* it was very difficult. The patients cried’* (Interview 1).

Most RNs experienced that it was bothersome using the PPE since it led to headache, dizziness, dehydration, warmth, and that these effects limited the amount of time spent with the patients, which affected the quality of care even more. Additionally, changing procedures was time consuming. This led to less time with patients in isolation or quarantine than compared to other patients. One RN working in the nursing homes described this as follows:

*‘Sometimes*,* you walked out of that door a little too quickly because I couldn’t bear to be there anymore. It was so hot and uncomfortable*’ (Interview 1).

However, in the ICUs, RNs experiences differed in that they had to stay in the isolated patient’s room for up to five hours without the possibility of leaving the room due to infection control. This led to more time with the patients and opportunities to talk and care for them in a greater extent than before, promoting the possibility to provide higher quality care.

On the other hand, RNs experienced that the PPE gave security, and thereby opportunity to be close to the patients physically. When using this in line with the guidelines, they trusted not to be infected, and therefore could be close to (touching) the patients. However, in the beginning of the pandemic RNs experienced a shortage of necessary equipment which then required the use of equipment against the normal guidelines and led to uncertainties regarding trust of the equipment. Both the uncertainties and the discomfort of using PPE affected the quality of care because the RNs were reluctant to be close to the patients if the patients needed them. A crucial milestone was also the COVID-19 vaccine which allowed RNs to be closer to the patients.

### Unpredictable workday

Being updated on the infection control guidelines was perceived as important to enable the RNs to provide quality care. RNs in both nursing homes and ICUs experienced an unpredictable workday due to constantly changing guidelines and restrictions due to infection control. This led to uncertainty about what to inform relatives coming to visit, for example:

‘…*there have been changes all the time*,* which have been a bit difficult to keep up with*,* actually*’ (Interview 4).

RNs strived to gain information about the pandemic, restrictions, and infection control guidelines. RNs experienced the importance of good leadership during the unpredictable work situation, who provided the RNs with the information needed and that was available:

‘*We had a very calm and restrained manager who gave us clear information all the time. So… there was no panic*,* I would say it was just learning*’ (Interview 16).

Also, an unpredictable workday was experienced by RNs in relation to what to expect from how the pandemic was developing. Seeing the media from other countries focusing on high numbers of patients with severe symptoms, many deaths and many RNs who were infected and died made an impact on the RNs. This led to feelings of insecurity, a state of constant alert and preparations in the units to care for the patients in the best way possible. For example, planning for a COVID unit in nursing homes, planning for admission of many more ICU patients than normal, planning for treatment guidelines and what to do to ensure the best care for the patients if they did not have enough oxygen, equipment, staff, or rooms. As one RN in the ICU expressed:

‘*We were told that we*,* possibly*,* should be able to care for 80 patients on [a] ventilator*,* which felt… completely unreasonable’* (Interview 12).

### The right person for the right assignment at the right time

A lack of access to qualified personnel was caused by both a high number of sick leaves among RNs and the infection control restrictions that caused RNs to stay home with minor symptoms. In nursing homes, RNs were relocated from other units or hired in, but it was common to hire assistant nurses, with or without training as assistant nurses or in palliative care, and without experience with the patient group and without knowledge of the patients in the ward, due to in the lack of available RNs. One RN stated that:

*It is important to have the resources to meet*,* both [for] patients and relatives. That we have enough heads and hands… not just that we are heads and hands*,* but that the heads who are here have the right competence to take care. It is important* (Interview 15).

The lack of RN competence was perceived as a threat to the quality of care. RNs experienced that being alone as a RN led to less time with patients, less person-centred care and the need to prioritising the most important. The lack of RN expertise also reduced the ability to consult colleagues or doctors or get help when needed, which led to longer waiting times for treatment and care, complicated routines such as double-checking medication and sometimes led to adverse events.

These challenges also led to an increased workload for the remaining RNs and uncertainties about being enough qualified RNs when coming to work:

‘…*when you’re left alone and there’s a lot to do…and you don’t get the help you need…there’s so much you might not get done…because of priorities*’ (Interview 8).

### Presence and absence of relatives and friends

The infection control guidelines led to restrictions for relatives and friends to be able to visit patients in hospitals and nursing homes, especially in the first phase of the pandemic when the only possibility for visiting patients was when patients were dying. RNs experienced the presence and absence of relatives and friends in different ways. When relatives and friends were absent, RNs experienced that this was difficult and heartbreaking in that patient lost valuable time with their relatives. Many of the RNs became emotionally affected by talking about this. In the absence of relatives, RNs perceived that they became—and should fulfil the role of—a substitute for the relatives to fulfil good palliative care.

*I have missed relatives. They were allowed to come visit at the end of life… it has been very*,* very sad that the patient have been alone for weeks… lost valuable time with their relatives. And relatives have lost valuable time with them…that has been one of the most difficult things during the pandemic…and that applies to both patients with and without COVID. Those without COVID didn’t get many visits either… So*,* it’s been a loss*,* and I think a loss for posterity*,* for those who have said goodbye to some of theirs… That they couldn’t be there at the end…* (Interview 13).

This was also perceived by the RNs to be a particularly hard time for the relatives. The RNs experienced relatives to be more anxious, worried and suffering because they could not visit their close ones. RNs differed in terms of whether relatives received sufficient support, regardless of the ward type. Some experienced that the relatives received less information and support, while others perceived that despite the abnormal situation, they managed to make relatives feel seen, acknowledged, and safe in providing them with necessary infection control equipment.

Some of the RNs perceived that the ward became less busy when the relatives were not present, and this led to more time and better care for the patients. Consequently, some units will continue with visiting hours after the pandemic. As one RN stated:

‘*Actually*,* I think we were able to do more and that we were able to take better care of the patient. They received better nursing and care without relatives’ present*’ (Interview 11).

When relatives were present, RNs experienced a new role of being a gatekeeper and watchman regarding protective equipment. They helped relatives dress correctly and reminded relatives of the infection control restrictions and rules. This was perceived as extended workload in an already very busy work situation. Also, RNs had to prioritise between relatives that could visit the dying patients due to the visiting restrictions, which was perceived as uncomfortable and hard. This new role was perceived as negative for some, especially those who had less work experience. RNs with long work experience were often asked to take this role, since they felt more confident. However, this new role was also experienced as a way of caring for relatives’ safety by informing and reassuring relatives regarding infection control, which was perceived positively by the RNs to provide quality support to the relatives.

### Situations that required creativity

The abnormal work situation due to the pandemic forced the RNs to be creative in their strive to maintain the quality of care. The isolating of patients with COVID led to difficulties in communication between the RNs in the patients’ rooms and colleagues, physicians, or other personnel, forcing creativity in new ways of communication. The limited possibility to communicate or discuss important issues with other personnel, double check patients’ medication and being alone with important decisions, was perceived by RNs to have been a serious threat to both the quality of palliative care and the safety of the patients. In the beginning of the pandemic, the RNs in the ICU communicated by writing messages on post-it notes in the windows. Also, alternative digital communication methods were used, e.g., walkie talkies, telephone and skype. The RNs also experienced that implementing digital patient curve was perceived to improve the communication, patients’ safety, and quality of care.

In addition, such digital communication platforms were used to enable communication between patients and relatives:

‘*We took iPad into the room so they could FaceTime…it was a very sick patient. Then the relatives could see the patient*,* and how it all looked’* (Interview 8).

In nursing homes, this creativity was present to enable patients and relatives to meet face to face by visiting through windows, terrasses or by driving the patients in their beds outside to meet their families.

*The patients who longed very*,* very much for their loved ones*,* then we drove them out… or they stood outside the window… We had many lovely moments… almost Romeo and Juliet… It was touching… At least those who were lucky enough to live in the two or three rooms there*,* which had a window…* (Interview 1).

Infection control and restrictions also led to challenges for physicians to visit the patients. This led to creativity in that the RNs arranged so that patients could meet the physicians digitally in a separate room.

The RNs emphasised that the pandemic led to a sense of being strong together in the face of a joint enemy. This sense of togetherness made them creative and flexible in providing the best possible palliative care to patients and their families. They felt that together they could manage and overcome the difficulties that lay ahead, as these quotes illustrate:

‘*In the beginning*,* as well*,* I almost felt a bit of a fighting spirit*,* now we really have to stand together and step forward*,’ (Interview 12) and, ‘*I have to say that we are flexible and go the extra mile to make it happen*’ (Interview 7).

## Discussion of the results

The RNs strived to achieve control to maintain the quality of palliative care despite the unpredictable work situation. RNs experienced that the quality depended on the situation (what), the perspective (who), the care context (where) and on the time perspective (when). Quality of care may be considered within the six dimensions, that the care services are: (a) safe, (b) effective, (c) patient-centred, (d) timely, (e) efficient and (f) equitable [10, 23]. However, quality of care may differ according to time, the cultural context, the perspective of those who define the quality of care (e.g., patients, RNs, leaders, policy makers) and the organisational level (individual, organisational or societal) [24, 25]. This is in line with the results of this study, that the perceptions of quality of care varied according to time, the care situation and care context and the perspective. Additionally, our study contributes with new knowledge to the field in that the RNs had various perceptions regarding how the quality of care was influenced by the COVID-19 pandemic; that the quality of care was preserved, lower or, in fact, higher than before the pandemic.

In this study the nursing care during the pandemic was perceived as challenging, which has been confirmed in previous studies [7, 17, 26]. However, many of these studies are related to nurses working on the frontline in the beginning of the pandemic as described in a review by Jackson and colleagues (2022).This study showed that working in the frontline affected nurses emotionally, physically, ethically and morally [7]. Few studies have investigated how these emotions affected the quality of care, especially related to palliative care, later in the pandemic and in differing care contexts. 

The participants experienced that the toolbox did not fit the challenges they faced during the pandemic. This toolbox was related to external factors such as lacking/missing guidelines and guidelines that did not apply for patients with COVID-19 but also internal factors that their experience and competence were insufficient for providing palliative care for this specific patient group. Guidelines for providing quality palliative care exist. Examples of such guidelines are a local and national palliative care plan, with instructions of medications in the last days of life, ‘The last days of life’ which is the Norwegian revised version of the Liverpool Care Pathway, to guide the care near the end of life and national guidelines which is a more extensive guideline for the whole palliative care trajectory [2]. Even care models to ensure person-centred care in the palliative phase have been developed [27]. These are all based upon the definition and goal of palliative care to relieve physical, psychosocial and existential suffering of patients, as well as support and care for their relatives [3]. However, patients with COVID needed a different approach/treatment than the patients they usually cared for. One example was the need for higher doses of morphine and prone positioning in the ICU. When the guidelines did not fit the patient’s situation or were lacking, the RNs became unsure about their competence in providing the best care for their patients. These uncertainties might be related to that none of the participants in this study had completed further education in palliative care. The reason for this might be that most of the participant were included from non-specialised palliative care contexts. One the other hand, five of the RNs had further education in cancer nursing care that in Norway includes training in palliative care. Previous studies are not congruent regarding how nurses experience their competence in providing quality palliative care. One previous study found that frontline nurses perceived they had sufficient competence to handle the challenges when facing this new patient group, but that graduated nurses perceived these challenges more stressful [7]. These findings contradict the results from a Norwegian report, which found nurses in community care experienced insecurity regarding the care of patients infected with COVID-19, especially related to pain relief in the beginning of the pandemic [6]. However, Jackson and colleagues’ results confirm that this may vary according to the care experience, in that more experienced nurses transferred their competencies towards this new patient group [7]. The results of this present study expand on the literature as the first to investigate quality of palliative care during the pandemic through RN competence and experience. Additionally, studies have previously found that the wards were lacking routines and guidelines to manage the new group of patients following the COVID-19 pandemic, especially in the beginning of the pandemic [8, 28]. This emphasised the importance of implementing a palliative care approach during a pandemic [29] and developing new guidelines to ensure the provision of the palliative care principles for this specific group of patients and for non-specialised palliative care contexts [5, 30].

The RNs in this present study experienced that using PPE led to social distance from patients and relatives, but—on the other hand—also gave opportunities for being close. For patients at the end of life, close contact with relatives and good communication are of outmost importance [31] and are prerequisites for providing quality palliative care together with holistic symptom control, a multidisciplinary approach and support for relatives [2]. The COVID-19 pandemic has led to comprehensive restrictions in all healthcare services, both in the specialist healthcare and in the community-based services [5]. Several studies have described how PPE has been experienced by nurses [7, 32] and how this has negatively affected the palliative care for the patients and relatives [33]. Examples are shortages of PPE, restrictions regarding visits from relatives and friends, preferred distance between peoples of 1–2 m and wearing facial masks and other equipment. These factors led to barriers in communication and reduced possibilities to fulfil the palliative care ideals/goals for patients and their relatives [26, 33]. Another study found RN’s fear of catching or spreading the virus [34] may affect the quality of care [2]. However, this present study contributes with new knowledge in that RNs also experienced positive aspects of wearing PPE, in that it enabled them to be close the patients and relatives, which enhanced the possibility to provide quality palliative care.

RNs in both nursing homes and ICUs experienced an unpredictable workday due to constantly changing guidelines and restrictions due to infection control. This led to a constant need for being updated on the latest guidelines to ensure high palliative care quality. These results have been confirmed in previous studies and reports [6, 18, 35]. The first phase of the pandemic was perceived as chaotic, stressful and lacking routines and guidelines [6]. However, later this was perceived differently with better preparedness to handle more patients being infected [6]. The results of this study indicated that this constant need to strive for being updated about the latest guidelines about infection preventions and restrictions was connected to the quality of care. Although few of the previous studies explicitly described how the quality of care was affected by the unpredictable workday, one study from a palliative care unit described how the nurses ‘armed themselves’ with information for the use of protection of the patients and their relatives, and themselves [18].

The lack of access to qualified personnel and RN competence, due to sick leaves and replacing RNs with other personnel with lower competence or not familiar with the ward or the patients, led to an increased workload to the remaining RNs and was perceived as a threat to the quality of care. The increased workload and replacement of RNs during the pandemic has been described in previous studies, e.g [6, 26, 33, 36, 37]. however, few studies described how this affected the RNs’ experiences of the quality of palliative care. One study from the hospital context found that the increased workload led the healthcare workers to prioritise between important aspects of palliative care and the quality of care decreased as a consequence [33]. Another study from the ICU context found that nurses in the ICU had negative experiences of replacing nurses from other units that did not have sufficient training and competence [37]. This emphasize the importance of leadership to assign the right person for the right assignment at the right time when replacing or relocating RNs, both in normal situations and in abnormal situations in the future such as future pandemics or crises.

The pandemic led to restrictions for relatives and friends to be able to visit patients in hospital and nursing homes. RNs in this present study experienced that the quality of palliative care was affected by the presence and absence of relatives and friends in different ways. Some of the RNs perceived this as difficult and heartbreaking on behalf of the patients and their relatives since they lost valuable time together, experienced loneliness and coped worse with their situation. To compensate, and thereby strive to fulfil the principle of quality palliative care, RNs perceived that they became—and should fulfil the role as—a substitute for the relatives. It has been described in previous studies from ICU and palliative care contexts that the visiting restrictions compromised the quality of family care [38], made end-of-life discussions more difficult [28], increased the burden on relatives [5], and that RNs experienced that they were not able to provide quality care in line with the palliative care ideals and standards [33]. Not being able to provide family care as usual, and according to the RNs care beliefs and standards, they felt distress and that the palliative care became inhuman [33]. Also, new roles were developed as RNs began to perceive themselves as extended family members advocating for the patients in the absence of families [33]. It has also been found that nurses missed opportunities to be flexible related to individual adjustments to visit restrictions [8]. The abnormal work situation due to the pandemic forced the RNs in this study to be creative in their strive to maintain the quality of care, using digital platforms to enable communication or arrange meetings with patients and relatives outdoors. This has been confirmed by previous studies that the pandemic led to extended use of digital platforms to enhance communication [6, 17, 33, 35]. However, critiques have been raised that digital communications had limitations and could not replace face-to-face contact [36], e.g., for patients with dementia and for dying patients. It would be useful to explore how to best use digital platform for these patient groups in future pandemics and crises.

In this study RNs were given a new role of gatekeeper/watchman regarding PPE and had to prioritise between which relatives could visit to ensure infection control and patients safety. The absence of relatives also made the ward less busy, which led to more time with the patients and better care for the patients. Consequently, some units will continue with visiting hours (restricted visiting as opposed to open visiting) after the pandemic. This has not been found in previous studies. The knowledge of the loneliness and suffering of patients and families that were not allowed to spent time together in the end of life has highlighted the importance of social relations between patients and relatives in palliative care and contradict the results in this study about maintaining restricted visiting hours. Wards with restricted visiting hours should consider these results before deciding to continue such practice.

Surprisingly, this study revealed that the pandemic also led to positive feelings of being strong together in facing a joint enemy. This feeling of togetherness made the RNs strong, creative, and flexible in providing the best palliative care for the patients and their families. A review about frontline nurses’ experiences similarly found that the pandemic led to an increased professional impact and enhanced professional identity [7]. Also, RNs and leaders from the nursing home context underscore that even if the pandemic has been challenging, the cooperation and feeling of unity amongst the staff has increased as well as new knowledge has been gained about infection control and digital communication [8].

### Methodological considerations

To report trustworthiness, the criteria of credibility, dependability and transferability are used [39]. In qualitative content analysis it is important to have variation and diversity in the material [21]. This requirement has been fulfilled because the participants varied in age and length of work experience. They all had experiences with palliative care during the pandemic from different contexts like intensive care units, palliative care units and the dementia care unit in a nursing home. Furthermore, by exploring in three different contexts, we achieved varied experiences.

The preunderstanding of the researchers may influence the trustworthiness of the results. The research group holds clinical competence and research experience from palliative care nursing and psychology. Credibility was ensured via continuous dialogue between the researchers to reach consensus regarding the content and labelling of the categories and theme to ensure that these were in accordance with the content of the interviews and the aim of this study. The authors discussed the interpretation until a consensus was reached.

Dependability refers to the degree to which data change over time [39]. The data in this study were collected from November 2021 to January 2022. The RNs were asked the same questions with adapted follow-up questions. The interview guide was developed and discussed by all participants in the research group. In addition, two pilot interviews were performed to assure the quality of the interview guide.

Transferability refers to how well the results can be transferred to other settings [21]. In qualitative research, the goal is not to generalise the results; instead, the results can be transferred to similar situations if they are recontextualised [39]. From the perspective of transferability, the background information of the participants, the context of the study and the analysis have been described in detail. The interviews were rich in content and described similar experiences, thereby creating a pattern that the authors found adequate to serve as a basis for the results. Even if the results were generated from the palliative care context, some of the results may reflect a more general situation in several healthcare contexts during the pandemic e.g. the experiences of unpredictable workday and challenges using PPE, and thereby be relevant and transferable to other settings.

To appraise the quality of the study, the COREQ checklist was applied.

One limitation of this present study could be the preponderance of women in the study (17 females and one male). On the other hand, this reflects reality in the nursing profession where there is a preponderance of women. Another limitation could be that there were six researchers who collected the data, this may affect variance in follow-up questions.

## Conclusion

Conclusions that can be drawn from the results are that although it was experienced as challenging to practice palliative care during the COVID-19 pandemic, the RNs had different experiences of how the quality of care was affected. Experiences varied from the quality of care being unchanged, to deteriorating, to being better than before the pandemic. RNs experienced that the challenging situation forced them to be creative and to strive for control to provide the best palliative care possible given the situation and resulted in a feeling of unity and togetherness among the staff. The results of this study may contribute to important knowledge for leaders, policy makers and nurses to learn from the COVID-19 pandemic and plan for future pandemics or crises.

### Relevance for clinical practice

The results of this study have underlined the importance to plan for and develop palliative care guidelines that applies for a pandemic situation and patients. These results especially shed light on factors related to the specific situation and care context, the perspectives of those involved and the time perspective of the pandemic, which leaders and policy makers may aim to optimize in the future.

The replacement of RNs with lower-qualified personnel or nurses who do not possess sufficient training or competence in respective specialities needed in the wards, palliative care or knowing the patients in the wards may affect the quality of palliative care and increase the workload for the remaining RNs. This emphasises the need for competence development and training programmes, especially for RNs with lower work experience and for replaced staff.

The results highlight the importance of sufficient access to PPE and knowledge that PPE may lead to social distance from patients and relatives, but—on the other hand—give opportunities for being close.

The knowledge of the loneliness and suffering of patients and family that were not allowed to spent time together in the end of life, and the importance of being creative to find alternative ways to communicate has emphasize the importance of social relations between patients and relatives in palliative care.

The pandemic led to positive feelings of being strong together to face a joint enemy. This feeling of togetherness made the RNs strong, creative, and flexible in providing quality palliative care. However, it is important that the RNs have the possibility to reflect upon the distressing situations to avoid burnout.

## Electronic supplementary material

Below is the link to the electronic supplementary material.

Supplementary Material 1

## Data Availability

The qualitative data generated and/or analysed during the current study are not publicly available due to the consent the respondent gave but are available from the corresponding author on reasonable request.

## Abbreviations

RN: Registered nurse
RNT: Registered nurse teacher
PPE: Personal protective equipment
ICU: Intensive care units
PAQC-19: Palliative Quality Care COVID-19
COREQ: Consolidated criteria for reporting qualitative research
