# Supplementary material for: ‘Striving to achieve control’. Registered nurses’ experiences of palliative care quality during the COVID-19 pandemic – a qualitative study
Source: BMC Palliat Care. 2025 Jan 23;24:21. doi: 10.1186/s12904-024-01644-8 (PMC11756187; doi:10.1186/s12904-024-01644-8)
Supplement: Supplementary file 1 — Supplementary Material 1 [file 12904_2024_1644_MOESM1_ESM.docx]

**Supplementary file 1. Interview guide.**

**Content of the interview guide for the study: ‘Striving to achieve control’. Registered nurses’ experiences of palliative care quality during the COVID-19 pandemic – a qualitative study**

What characterises (good) person-centred palliative care for you?

What is your experience in providing palliative care to life - threatening ill and dying patients during the COVID-19 pandemic?

- Can you please tell me about a situation where care was good?

Which factors promoted the possibility to provide good person-centred palliative care during the pandemic?

- Can you please tell me about a situation where care was not so good?

Which factors inhibited the possibility to provide good person-centred palliative care during the pandemic?

Follow-up questions were used, such as: Can you please explain more about that…?
